# An Efficient Preparation of Mulberroside A from the Branch Bark of Mulberry and Its Effect on the Inhibition of Tyrosinase Activity

**DOI:** 10.1371/journal.pone.0109396

**Published:** 2014-10-09

**Authors:** Shu Wang, Xian-Ming Liu, Jian Zhang, Yu-Qing Zhang

**Affiliations:** 1 Silk Biotechnology Laboratory, School of Basic Medical and Biological Sciences, Soochow University, Dushuhu Higher Edu. Town, Suzhou, P R China; 2 College of Pharmaceutical Sciences, Soochow University, Dushuhu Higher Edu. Town, Suzhou, P R China; Bhabha Atomic Research Centre, India

## Abstract

A bioactive ingredient in an ethanol extract from the branch bark of cultivated mulberry Husang-32 (*Morus multicaulis* Perr.) was isolated using a macroporous resin column. The primary component, which was purified by semi-preparative high-performance liquid chromatography diode array detection (HPLC-DAD), was identified as mulberroside A (MA) by liquid chromatograph-mass spectrometer (LC-MS), ^1^H and ^13^C nuclear magnetic resonance (NMR) spectra. In total, 4.12 g MA was efficiently extracted from one kilogram of mulberry bark. The enzymatic analysis showed that MA inhibited the generation of dopachrome by affecting the activities of monophenolase and diphenolase of tyrosinase *in vitro*. This analysis indicated that MA and oxyresveratrol (OR), which is the the aglycone of mulberroside A, exhibited strong inhibition of the monophenolase activity with IC_50_ values of 1.29 µmol/L and 0.12 µmol/L, respectively. However, the former showed weaker inhibitory activity than the latter for diphenolase. For the monophenolase activity, the inhibitory activity of MA and OR was reversible and showed mixed type 1 inhibition. Additionally, the inhibition constant KI (the inhibition constant of the effectors on tyrosinase) values were 0.385 µmol/L and 0.926 µmol/L, respectively, and the KIS (the inhibition constants of the enzyme-substrate complex) values were 0.177 µmol/L and 0.662 µmol/L, respectively. However, MA showed competitive inhibition of diphenolase activity, and *K_I_* was 4.36 µmol/L. In contrast, OR showed noncompetitive inhibition and *K_I_* = *K_IS_* = 2.95 µmol/L. Taken together, these results provide important information concerning the inhibitory mechanism of MA on melanin synthesis, which is widely used in whitening cosmetics.

## Introduction

Tyrosinase, which is a polyphenol oxidase, is the key enzyme in melanin synthesis and possesses monophenolase and diphenolase activity. This enzyme can catalyse two distinct reactions: the oxidation of L-tyrosine to L-dihydroxyphenylalanine (L-DOPA) (first reaction) and the oxidation of L-DOPA to dopaquinone (second reaction). Then, dopaquinone, through a non-enzyme-catalysed process, is transformed into leukodopachrome (third reaction). Leukodopachrome is oxidised into dopachrome by a dopaquinone (fourth reaction), which is an extremely fast and non-enzyme-catalysed process. Then, dopachrome is transformed to melanin through a series of chemical- and enzyme-catalysed reactions. The process of tyrosinase catalysing L-tyrosine to dopachrome shows that dopachrome synthesis can be suppressed when any of the steps are inhibited. Additionally, not all substances that can inhibit the formation of dopachrome are tyrosinase inhibitors, for example, thymol [1].

Mulberry (Morus) trees, belongs to the family of Moraceae, have been widely cultivated in several sericulture countries (e.g. China, Korea, Japan and so on.). In fact, early in 2002 the Chinese Ministry of Health declared that the mulberry leaf and mulberry (fruit) were not only food, but also drugs; and the root bark (*Cortex Mori* albae) and branch were health food especially the root was widely used in the folk prescription of traditional Chinese medicine. Modern pharmacological study proves that the aqueous or methanol extracts of *Cortex Mori* have anti-inflammatory, hypoglycemic, hypotensive, anticarcinogenic and anti-microbial activities; and the chloroform and alkali extracts have the obvious anti-inflammation and anti-asthmatic activities.

**Figure 1:**
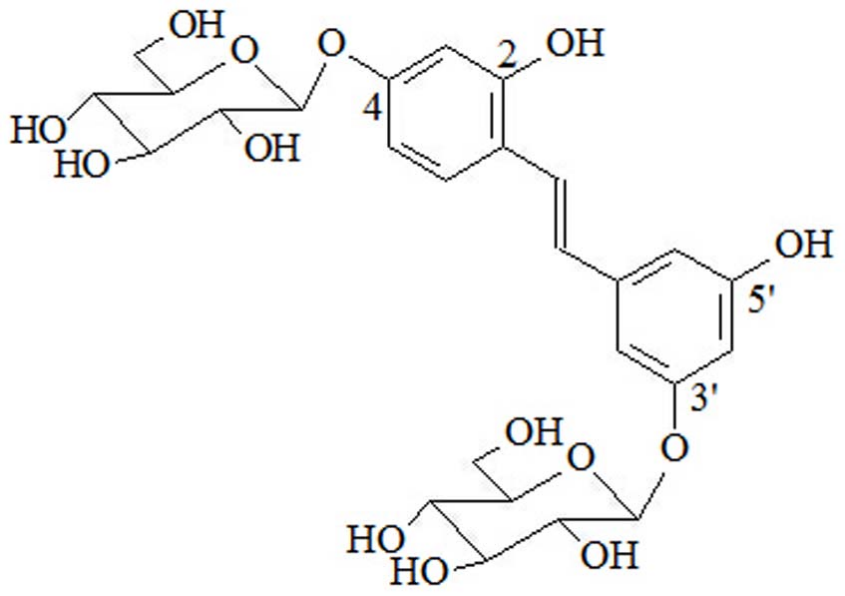
Structure of mulberroside A. (Oxyresveratrol-4-O-b-D-glucopyranosyl-3′-O-b-D-glucopyranoside or 2, 4, 3′, 5′-tetrahydroxystilbene).

Mulberroside A (MA) is an oxyresveratrol diglycopyranoside, where 4-OH and 3′-OH are substituted by two glucose molecules (Fig. 1). MA has *cis* and *trans* configurations. Early in 1986, this compound was first isolated from the acetone extract of Morus lhou Koidz root. After gavaging with aqueous extracts of mulberry root, Qiu et al. found that the stilbenes absorbed by rats were mostly MA [2], [3]. MA could also be separated from the alcohol extract of *Cortex mori* [4], and 1.346% MA was obtained from the root using ultrasonic extraction [5]. The tyrosinase inhibitory activity of MA from the root of *Morus albae* was greatly enhanced by the bioconversion process [6]. In addition, the ethyl acetate fraction of the alcohol extract from leaves [7] and the monomer separated from twigs [8], 2-oxyresverstrol, also had strong tyrosinase inhibitory activity. The deglucosylated form of MA had more potent tyrosinase inhibitory activity than its glycosylated form [9], and the IC_50_ value decreased from 42.06 µmol/L to 15.15 µmol/L. Kim [10] et al. determined the kinetics and mechanism for the inhibition of mushroom tyrosinase, which was reversibly inhibited by oxyresveratrol (OR) as a non-competitive inhibitor, with L-tyrosine as the substrate. Another study showed that the tyrosinase inhibitory activity of OR (0.49 µmol/L) was approximately 110-fold higher than that of MA (53.6 µmol/L), which was isolated from the ethanol extract of the roots of *Morus alba*, and the kinetics showed MA to be a competitive inhibitor of mushroom tyrosinase, with L-tyrosine and L-DOPA as substrates [6]. A mixture of OR and MA separated from the alcohol extract of twigs showed strong inhibition of melanin synthesis [6], [11]. Moreover, a decade later, a related medicinal analysis revealed that MA, which was extracted from the mulberry root, had antitussive, anti-asthmatic [12], expectorant [13], anti-diabetic [14], cell protection [15], [16], anti-oxidative, hepatoprotection [17], [18] and other pharmacological effects.

The above-mentioned research concerning the separation, purification, and identification of MA and its tyrosinase inhibition were primarily identified from the mulberry root. There is only rare systematic research and analyses regarding the extraction and separation of mulberry branches, which are often used as agricultural waste or as firewood. Thus far, there is a lack of systemically studies and analyses concerning the mechanism of the strong inhibitory action of MA on the monophenolase and diphenolase activities of the mushroom tyrosinase. In this study, we extracted an alcohol solution from *Ramulus mori* of the cultivated mulberry (Husang No. 32) *Morus multicaulis* Perr. Using a macroporous resin column for adsorption and for separation and then purification by reversed-phase high-performance liquid chromatography (RP-HPLC), we obtained much MA monomer. Next, we systemically studied and analysed the mechanism of the inhibition activity of MA on the mushroom tyrosinase.

## Materials and Methods

### 2.1 Plant material

Fresh one-year-old branches of cultivated mulberry (Husang No. 32) from *Morus multicaulis* Perr. were collected from the mulberry field of Soochow University in November 2010.

### 2.2 Preparation of the mulberry branch bark ethanol extract

The ethanol extract of mulberry branch bark was prepared by a previously described method [19], with slight modifications. The bark was peeled from the mulberry branches, then air-dried and milled. The bark powder was repeatedly extracted with an 80% ethanol solution under reflux 3 times, and the insoluble material was removed by filtration and by centrifugation. The resulting extracts were combined and concentrated under reduced pressure to obtain a residue and lyophilised into powders.

### 2.3 Isolation on a macroporous resin column

A weighed amount of the branch bark powder was dispersed in water, added to a D101 macroporous resin chromatography column (Shanghai Blue Season Science and Technology Development Co., Ltd.) and eluted with a gradient elution of ethanol-water (EtOH-H_2_O, 0%, 30%, and 100%). The fractions were concentrated and lyophilised into powders.

### 2.4 RP-HPLC for analysis and preparation

The experimental conditions of RP-HPLC for analysis and semi-preparation were as follows:

A Shimadzu LC-20A HPLC pump connected to a Shimadzu VP-ODS column (250×4.6 mm) was used to analyse the samples. All the sample used was precisely formulated into a solution of 0.5 mg/ml. Mobile phases A (0.4% aqueous acetic acid) and B (acetonitrile) were prepared according to the following procedure: initial 0-20 min, linear change from A/B = 95:5 (v/v) to A/B = 85:15 (v/v); 20–30 min, linear change from A/B = 85:15 (v/v) to A/B = 80:20 (v/v); 30–35 min, linear change from A/B = 80:20 (v/v) to A/B = 45:55 (v/v) and then held for 5 min. The mobile phase flow-rate was set at 1.0 mL/min, and aliquots of 10 µl were injected for analysis. The detection wavelength was 324 nm, scanning from 200 nm to 600 nm. All analyses were performed at room temperature.

The preparation of MA standard curve was as follows. In total, 10 mg of MA standard was dissolved in water and then formulated into a solution of 1.0 mg/mL. Samples of 2.0, 4.0, 6.0, 8.0, 10.0, 12.0, and 14.0 µL of MA standard solution were injected into the HPLC system. A standard curve was drawn, with the peak area as the vertical axis (*y*) and with the standard injection volume (µg) as the abscissa (*x*).

The sample preparation was performed using a Semi-Preparative HPLC (Shimadzu LC-6A) equipped with a DAD detector (SPDM20A). The semi-preparative column consisted of a Shim-pack Prep-ODS (H) column (250×20 mm) fitted with a guard module (Shim-pack G-ODS). Mobile phase A was 0.4% aqueous acetic acid, and mobile phase B was acetonitrile (Sinopharm Chemical Reagent Co., Ltd). The detection wavelength was 324 nm, scanning from 200 nm to 600 nm. All preparations were performed at room temperature. The gradient program used was as follows: 0–30 min, linear change from A/B = 93:7 (v/v) to A/B = 80:20 (v/v); 30–40 min, linear change from A/B = 80:20 (v/v) to A/B = 50:50 (v/v) and then held for 10 min. The flow rate was 5.0 mL/min, and aliquots of 5.0 mL were injected. The peak corresponding compound was collected at the retention time of 33.148 min and then concentrated and lyophilised into powders.

### 2.5 LC-MS analysis

The liquid chromatograph-mass spectrometer (LC-MS) conditions were as follows: a Q-Trap 2000 LC/MS/MS electro-spray ionisation source (ESI source) from ABI Company; an Agilent 1100 LC system; a Shimadzu Shim-Pack VP-ODS (250;mm×2.0 mm) chromatographic column, a column temperature of 18°C; mobile phase: 10% acetic acid aqueous solution:acetonitrile  = 60:40 (v/v); Enhanced Mass Spectrometry (EMS ^(+)^) scanning, 380°C; DP: 25; CUR: 30; G1: 40; G2: 35; EP: 10; CE: 10.

### 2.6 NMR analysis

The lyophilized powder of the sample was dissolved using 0.5 mL deuterated methanol (CD_3_OD) and 0.5 mL deuterated dimethyl sulphoxide (CD_3_SOCD_3_), respectively. The carbon spectrum (^13^C-NMR) and hydrogen spectrum (^1^H-NMR) were measured using a 300 MH_Z_ superconductive nuclear magnetic resonance (NMR) spectrometer.

### 2.7 Determination of tyrosinase activity inhibition

#### 2.7.1 The effect on the total process of dopachrome synthesis

The effect of tyrosinase (Worthington, USA) on the catalysis of L-tyrosine to dopachrome was determined using the method described by Hiroki [1], with slight modifications. The total reaction volume was 3 mL. First, 2.8 mL of 2.0 mM L-tyrosine was incubated at 30°C for 5 min, and then 100 µL of 1.0 µM sample solution and 100 µL of 2.0 mg/mL enzyme solution were added and mixed. The change in the absorption spectrum was determined using a Hitachi U3000 UV-visible spectrophotometer at 30°C isothermal conditions. The scanning wavelength ranged from 250 nm to 600 nm. The scanning frequency was 2 min, and the scanning time was 12 min. Thus, the changes in the absorption spectrum over time were obtained.

#### 2.7.2 The effect of the enzyme-catalysed process on dopachrome synthesis

The effect of the enzyme-catalysed process was determined using a previously described method [1], with slight modifications. In total, 100 mg N-acetyl-L-tyrosine was dissolved using NaH_2_PO_4_-Na_2_HPO_4_ temporary buffer solution (pH 6.8), and the volume was set to 100 mL. In total, 0.25 mg/mL enzyme solution and a certain concentration of the sample solution were prepared. All the solutions were filtered using a syringe filter with an aperture of 0.22 µm. The components of the reaction system were as follows: 80 µL N-acetyl-L-tyrosine was mixed with 10 µL sample solution and incubated at 30°C for 5 min, and then 10 µL enzyme solution was added. During the period from 0–60 min, 20 µL mixed solution was injected into the HPLC system every 10 min to detect changes in N-acetyl-L-tyrosine. The HPLC conditions were as follows: a Shimadzu HPLC pump equipped with a Shimadzu 9082652 VP-ODS column (250 mm×4.6 mm). The detection wavelength was 275 nm. The injection volume was 10 µL, and the flow-rate was 1.0 mL/min. The mobile phases were A (water) and B (acetonitrile), which were prepared according to the following linear procedure: 0–8 min, A/B = 85:15 (v/v). All analyses were performed at room temperature.

#### 2.7.3 The effect of the non-enzyme-catalysed process on dopachrome synthesis

The effect of the non-enzyme-catalysed process was determined by a previously described method [1], with slight modifications. The HPLC conditions were as follows: a Shimadzu HPLC pump equipped with a Shimadzu VP-ODS column (250 mm×4.6 mm). The detection wavelengths were 245 nm and 280 nm. The injection volume was 20 µL, and the flow-rate was 1.0 mL/min. The mobile phases were A (0.4% aqueous acetic acid) and B (acetonitrile), which were prepared according to the following linear procedure: 0–10 min, A/B = 90:10 (v/v).

### 2.8 Determination of the tyrosinase inhibition mechanism

#### 2.8.1 The inhibition of the tyrosinase monophenolase and diphenolase activity

The measurement of the sample's inhibitory activity on monophenolase activity was based on 0.5 mmol/L L-tyrosine as the substrate [20]. The total reaction volume was 3.0 mL, which included 2.8 mL substrate solution, 100 µL different concentrations of the sample solutions and 100 µL mushroom tyrosinase solution. The substrate and enzyme solution were dissolved in 50 mmol/L NaH_2_PO_4_-Na_2_HPO_4_ temporary buffer solution (pH 6.8), with a substrate concentration of 0.5 mmol/L and with an enzyme solution concentration of 1.0 mg/mL. The samples were dissolved in DMSO. First, 100 µL sample solution was added, then 2.8 mL substrate solution, which was pre-incubated at 30°C, was added, and last, 100 µL enzyme solution was added and immediately mixed. The curve was drawn using real-time tracking of the optical density change over time at 475 nm. The measuring instrument was a Hitachi U3000 UV-visible spectrophotometer. The enzyme activity was obtained from the slope of the line. The sample concentration-response curve was plotted as the inhibition rate of samples to the enzyme against the concentrations of effectors. The IC_50_ value, which represented the concentration of sample at which 50% of the tyrosinase activity was inhibited, was obtained from the curve.

The measurement of the sample's inhibitory activity on diphenolase activity was based on 0.5 mmol/L L-DOPA as the substrate. The experimental procedure and calculations were identical to those above-mentioned procedures and calculations.

#### 2.8.2 The inhibition mechanism of the tyrosinase monophenolase or diphenolase activity

The concentration of the substrate was fixed in 3.0 mL of 50 mmol/L NaH_2_PO_4_-Na_2_HPO_4_ temporary buffer solution (pH 6.8); however, the amount of enzyme was changed to determine the effects of different concentrations of sample on the reaction of mushroom tyrosinase catalysing L-tyrosine or L-DOPA as substrates. Mapping the reaction rate in terms of the amount of enzyme determined whether the inhibition was a reversible reaction. If the lines all passed through the origin, then reversible inhibition was demonstrated. However, if the lines were parallel, then irreversible inhibition was demonstrated.

And the inhibition type and inhibition constant were determined as follows. The concentration of the enzyme solution was fixed in 3.0 mL of 50 mmol/L NaH_2_PO_4_-Na_2_HPO_4_ temporary buffer solution (pH 6.8); however, the amount of substrate (L-tyrosine or L-DOPA) was changed to determine the effects of different concentrations of effectors on the reaction of mushroom tyrosinase catalysing the substrate. Using the Lineweaver-Burk method [6], the curve was plotted as the reciprocal of the reaction rate 1/V against the reciprocal of the substrate 1/[S] to determine the type of inhibition. If the lines intersected on the *y*-axis, then competitive inhibition was indicated. If the lines intersected on the *x*-axis, then noncompetitive inhibition was indicated. If the lines intersected in the first or fourth quadrant, then this inhibition was mixed type 1 or 2. If the lines were parallel, then anticompetitive inhibition was indicated. The inhibition constant of the effectors on tyrosinase (*K_I_*) and the inhibition constants of the enzyme-substrate complex (*K_IS_*) were obtained by a secondary plot of the concentration of the effectors against the slope of the line and the *y*-axis intercept, respectively.

## Results

### 3.1 The preparation of Mulberroside A

In total, 2.0 kg powder as treated using the previously described method. Finally, 350.68 g ethanol extract was obtained. The content of the ethanol extract was 17.5% of the powder. Then, the ethanol extract was dissolved in water at 2%∼5% concentrations and loaded onto a column packed with D101 macroporous resin. Following D101 macroporous resin column chromatography, the ethanol extract was divided into three parts: 259.19 g for the water elution fraction (W), 76.59 g for the 30% ethanol elution fraction (E30) and 5.65 g for E100. The results suggested that the bark ethanol extract was primarily composed of W (73.91%) and E30 (21.84%) and that the volume of E100 was small (1.61%). This HPLC analysis showed that the composition of E30 was relatively simple; therefore, subsequent experiments were focused on E30.

### 3.2 Isolation and Purification

**Figure 2:**
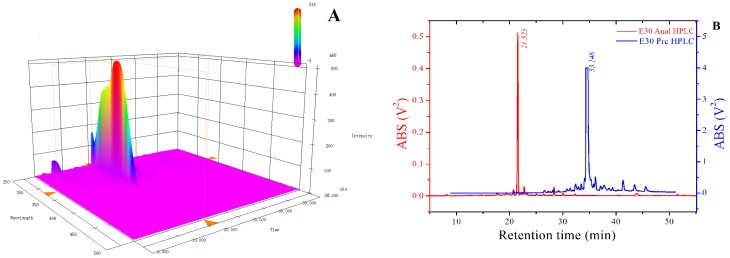
3D (A) and 2D profiles (B) of E30 fraction on analytic and prepared HPLC-DAD.

Using RP-HPLC to analyse E30, the analysis of the 3D chromatogram (Fig. 2(A)) demonstrated that the composition of E30 was relatively simple, and at the retention time of 21.52 min, E30 had a single strong peak corresponded to the main ingredient of E30. In the 3D chromatogram, the maximum absorption peak of the main ingredient was at 324 nm. To have a clearer view of the separation efficiency, we transferred the 2D chromatogram at the 324 nm wavelength from the 3D chromatogram (Fig. 2(B), blue line). As shown in Fig. 2(B), the retention time of 21.52 min showed an extremely sharp peak. The separation efficiency was excellent and suitable for use in the semi-preparative HPLC for further separation and for purification. The red line shown in Fig. 2(B) denotes the chromatogram of the main ingredient of E30 using the semi-preparative HPLC. The fraction of the peak was collected, concentrated and lyophilised to obtain the purified compound, which had a total weight of 8.24 g.

### 3.3 RP-HPLC-DAD analysis

**Figure 3:**
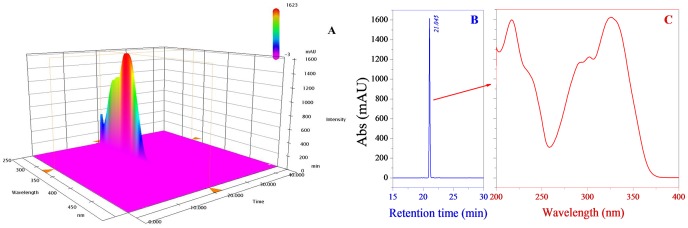
3D(A),2D(B) profiles on HPLC and UV-visible spectra(C)of MA.

There were no other interfering peaks, except at the retention time of 21.05 min in the 3D chromatogram (Fig. 3A), which showed that the compound obtained by semi-preparative HPLC was of high purity. Transferring the 200–500 nm UV-visible scan spectra from the 3D chromatogram (Fig. 3C) showed that the compound had a maximum absorption value at 324 nm. The 2D chromatogram at 324 nm, which was transferred from the 3D chromatogram (Fig. 3B), also showed that a single peak was only obtained at the retention time of 21.05 min. This monomer was used in the following NMR analysis.

### 3.4 LC-MS, NMR (^1^H, ^13^C spectrum) Identification

The compound obtained was identified using MS and NMR.

MS spectra showed the following positive ion ESI-MS m/z values: 591.2 [M+Na]^+^, 569.2 [M+H]^+^, 406.9 [M-162]^+^, 245.0 [M-162-162+H]^+^, and showed that the molecular weight of the compound was 568.2.

^1^H-NMR (CD_3_OD, 300 MHz) δ: 7.34 (1H, d, J = 9.3 Hz, H-6), 7.22 (1H, d, J = 16.5 Hz, α-H), 6.84 (1H, d, J = 16.5 Hz, β-H), 6.68 (1H, brs, H-6′), 6.54 (1H, m, H-5), 6.53 (1H, m, H-2′), 6.49 (1H, m, H-3), 6.36 (1H, brs, H-4′), 4.79 (1H, d, J = 7.5 Hz, C-4(glc)H-1″), 4.79 (1H, d, J = 7.5 Hz,C-3′ (glc)H-1′″), and 3.85–3.21 (12H, m). Anomeric H signals in the 3.85–3.21 (12H, m) are overlapping signals. The original NMR spectrum has been added as Figure S1.

^13^C-NMR (CD_3_OD, 75 MHz) δ: 120.2 (C-1), 157.1 (C-2), 104.8 (C-3), 160.4 (C-4), 109.3 (C-5), 128.4 (C-6), 127.6 (β-C), 124.9 (α-C), 141.9 (C-1′), 107.2 (C-2′), 159.5 (C-3′), 103.9 (C-4′), 159.5 (C-5′), 108.1 (C-6′), 102.0 (C-4(glc)C-1″), 74.8 (C-2″), 77.9 (C-3″), 71.3 (C-4″), 78.1 (C-5″), 62.4 (C-6″), 102.3 (C-3′ (glc)C-1′″), 74.9 (C-2′″), 77.9 (C-3′″), 71.3 (C-4′″), 78.1 (C-5′″), and 62.5 (C-6′″). The original NMR spectrum has been added as Figure S2.

^1^H-NMR (CD_3_SOCD_3_, 400 MHz) δ: 9.80 (1H, s, OH), 9.38 (1H, s, OH),7.44 (1H, d, J = 8.4 Hz, H-6), 7.22 (1H, d, J = 16.4 Hz, α-H), 6.94 (1H, d, J = 16.4 Hz, β-H), 6.64 (1H, brs, H-6′), 6.58 (1H, m, H-2′), 6.52 (1H, d, J = 8.4 Hz, H-5), 6.55 (1H, m, H-3), 6.35 (1H, brs, H-4′), 4.79 (1H, d, J = 7.2 Hz, C-4(glc)H-1″), 4.79 (1H, d, J = 7.2 Hz,C-3′ (glc)H-1′″), 5.24 (2H, dd, J = 10.0 Hz, 4.4 Hz), 5.10 (4H, m), 4.51 (2H, d, J = 6.4 Hz), 3.70 (2H, m), 3.50 (3H, m), and 3.18–3.30 (7H, m). Both anomeric H signals in the 3.50 (3H, m) and 3.18–3.30 (7H, m) are overlapping signals. The original NMR spectrum has been added as Figure S3.

By comparing the ^1^H-NMR (CD_3_OD), ^13^C-NMR and ^1^H-NMR (CD_3_SOCD_3_) data with the literature ^9, 16^, the compound was identified as oxyresveratrol-4-O-β-D-glucopyranosyl-3′-O-β-D-glucopyranoside or mulberroside A (MA). Its molecular formula is C_26_H_32_O_14_, and the structure is shown in Fig. 1.

### 3.5 The inhibition of MA on dopachrome synthesis

**Figure 4:**
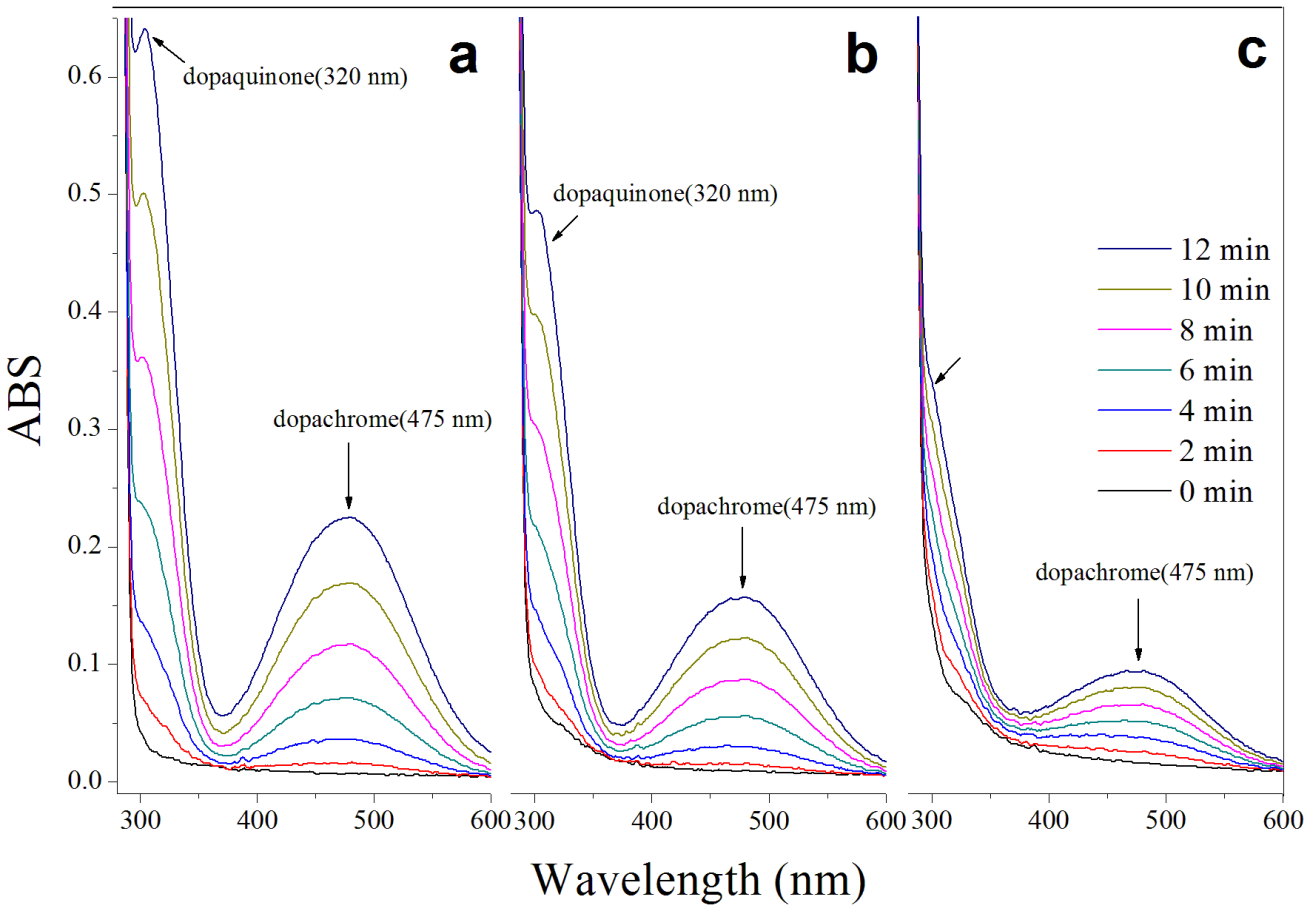
Consecutive UV-Vis spectra obtained in the oxidation of 0.5 mmol/L L-tyrosine by mushroom tyrosinase in the absence sample (a), in presence of 2 mmol/L mulberroside A (b) or oxyresveratrol (c) for 12 min. Spectra scans were repeatedly at a speed of 300 nm/30 s in 2 min intervals.

When no inhibitor was added, tyrosinase continuously catalysed L-tyrosine to dopaquinone and to dopachrome during the period from 0–12 min (spectral scanning every 2 min); thus, the contents of dopaquinone and dopachrome in the reaction system rose (Fig. 4a). Additionally, the results are shown for when 2.0 µmol/L MA and 2.0 µmol/L OR were added (Fig. 4b and Fig.4c). Compared with no inhibitor added, dopaquinone and dopachrome also increased with time; however, the rate slowed down, which indicated that MA and OR inhibited the formation of dopachrome. The effects of OR on the inhibition of tyrosinase have been previously studied; however, there have only been a few studies concerning MA. Whether MA and OR can influence the third reaction of dopachrome synthesis has not been reported, and our next experiment was designed to determine whether this influence occurred.

### 3.6 The effect MA on tyrosinase activity (monophenolase and diphenolase activity)

**Figure 5:**
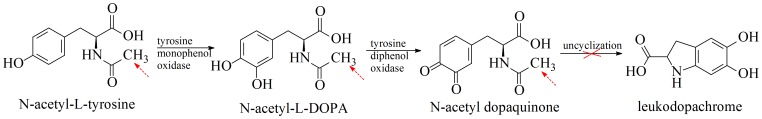
Using N-acetyl-L-tyrosine as a substrate, because of the presence of an acetate group on the N-group of an amino acid, intracyclization of dopaquinone to leukodopachrome is blocked.

N-acetyl-L-tyrosine was used instead of L-tyrosine as the substrate for mushroom tyrosinase (Fig. 5) because the monophenolase activity of the mushroom tyrosinase can catalyse the conversion of N-acetyl-L-tyrosine to N-acetyl-L-DOPA and because diphenolase can catalyse N-acetyl-L-DOPA to N-acetyl-dopaquinone. Due to an acetate group on the amino of N-acetyl-dopaquinone, cyclisation cannot occur; thus, the reaction was blocked. Therefore, changes in N-acetyl-L-tyrosine in the reaction system can directly reflect the influence of the sample on mushroom tyrosinase activity. If the sample added to the reaction system cannot inhibit the activity of mushroom tyrosinase, then the content of N-acetyl-L-tyrosine will gradually decrease over time (the peak height of N-acetyl-L-tyrosine that can be observed in the HPLC chromatogram will decrease); however, if the activity of mushroom tyrosinase is inhibited, then the content of N-acetyl-L-tyrosine will not change over time (the peak height of N-acetyl-L-tyrosine will not change or will decrease extremely slowly in the HPLC chromatogram).

**Figure 6:**
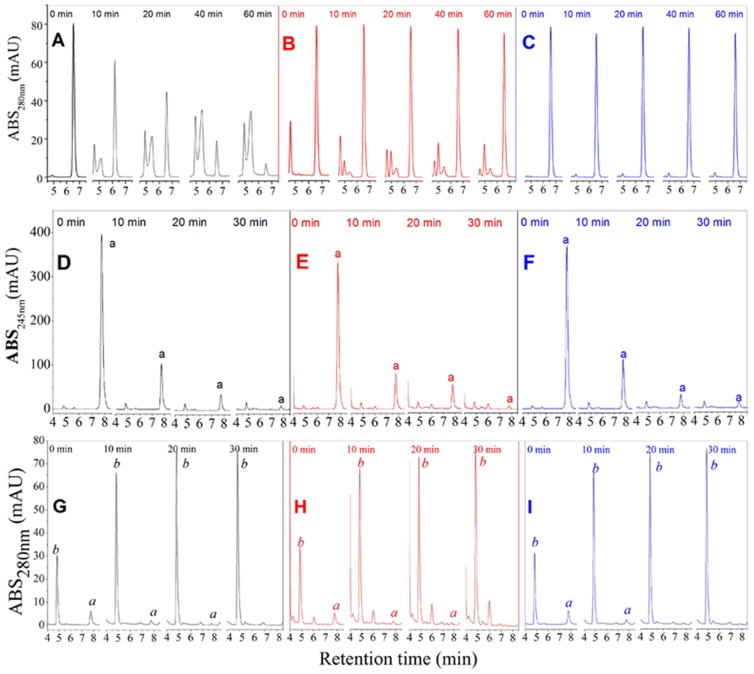
The change in the content of N-acetyl-L-tyrosine as a substrate for tyrosinase in the absence of sample (A), in the presence of 175.9 µmol/L MA (B) or oxyresveratrol (C) during 60 min. HPLC analysis of the redox reaction of p-benzoquinone and L-DOPA in the absence of sample (D), or in the presence of mulberroside A (E), or in the presence of oxyresveratrol (F) at 245 nm. HPLC profiles of the redox reaction of p-benzoquinone and L-DOPA in the absence of PBS (G), mulberroside A (H) or oxyresveratrol (I) at different reaction times of 0, 10, 20 and 30 min.

Fig. 6A, B, and C show the changes of N-acetyl-L-tyrosine content in the reaction system when N-acetyl-L-tyrosine, as a substrate, was added to the buffer solution, to 175.9 µmol/L MA, and to 175.9 µmol/L OR, respectively. The retention time of N-acetyl-L-tyrosine in the column was 6.5 min. Without inhibitors, the figures show that N-acetyl-L-tyrosine decreased by 44.35% after 20 min, by 60.53% at 40 min, and by 73.07% after 1 h. These results showed that N-acetyl-L-tyrosine was transformed by tyrosinase in the reaction system. However, when added to 175.9 µmol/L MA and OR, the content of N-acetyl-L-tyrosine did not change over the period from 0–60 min. These results showed that MA and OR inhibited the activity of tyrosinase, resulting in no change in N-acetyl-L-tyrosine content throughout the reaction.

### 3.7 The inhibition of MA on dopaquinone to dopachrome

To measure whether the samples affected the second stage of the dopachrome synthesis, we used L-DOPA and *p*-benzoquinone to substitute leukodopachrome and dopaquinone to simulate the non-enzymatic process. L-DOPA and *p*-benzoquinone were converted to dopaquinone and to hydroquinone by a redox reaction. HPLC was used to detect the changes in *p*-benzoquinone and hydroquinone contents over time in the reaction system, and the results are shown in Fig. 6D-F. The maximum absorption peaks of *p*-benzoquinone and hydroquinone were at 245 nm and 280 nm, respectively, and the retention times were 7.76 min and 4.86 min in the Shimadzu VP-ODS column (250 mm×4.6 mm), respectively. Fig. 6D–F shows the HPLC chromatogram at 245 nm wavelength, and Fig. 6D shows the change in *p*-benzoquinone when no samples were added to the system. Within 10 min, *p*-benzoquinone decreased by 75%, and at 20 min, *p*-benzoquinone decreased by 90.62%. Fig. 6E and F represent the results when MA and OR were added. The results indicated that the declining trend in *p*-benzoquinone content did not change and was not suppressed by the addition of MA and OR. As shown in Fig. 5G–I, the content of hydroquinone also did not change at 280 nm when compared with the analysis when the samples were not added. These results showed that the redox reactions of L-DOPA and *p*-benzoquinone were not influenced by MA and OR. Therefore, MA and OR do not play a role in the conversion of dopaquinone to dopachrome, which further illustrates that MA is a real tyrosinase inhibitor, similar to OR, and has inhibitory activity on both monophenolase and diphenolase.

### 3.8 The inhibition of MA on monophenolase activity of tyrosinase

**Figure 7:**
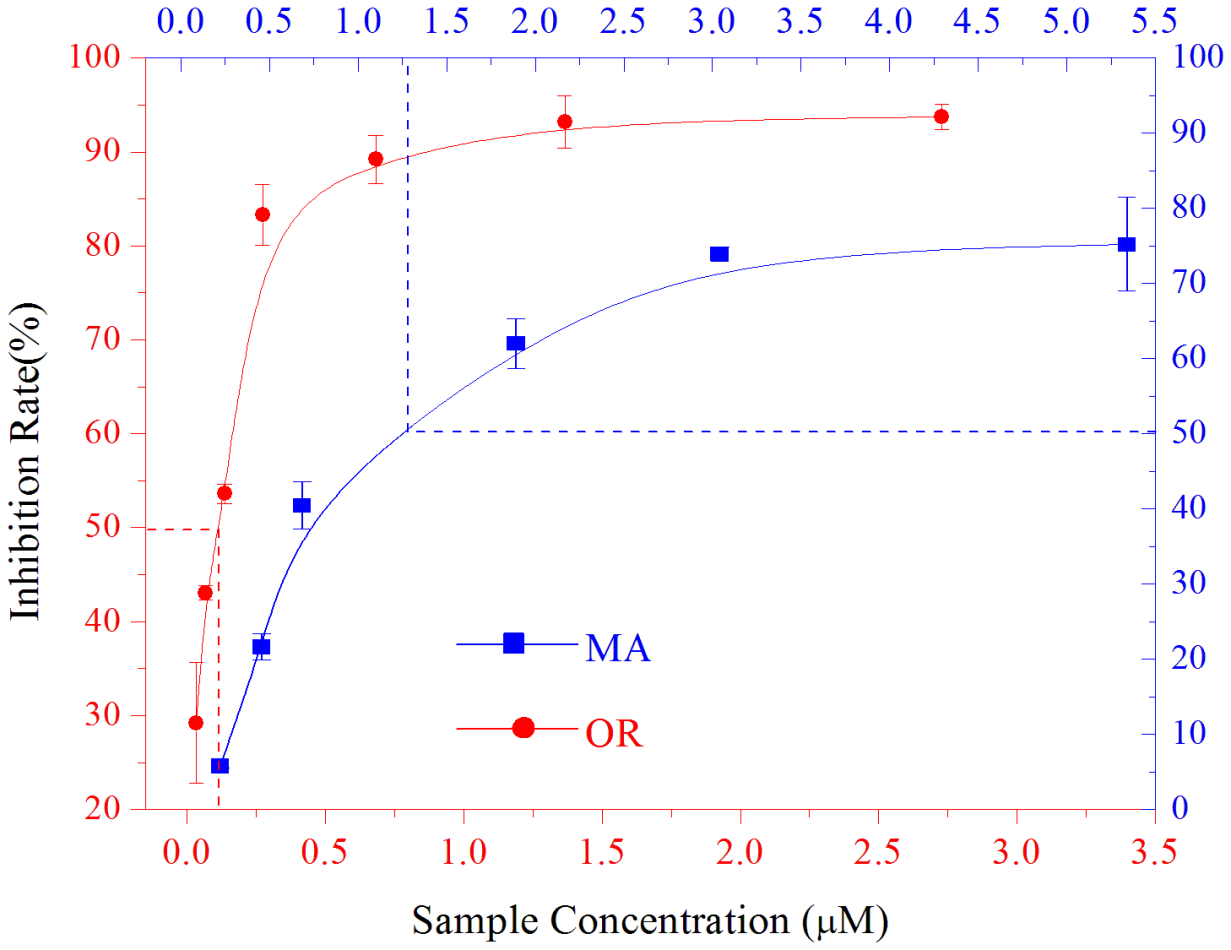
Inhibition effects of mulberroside A (blue line) and oxyresveratrol (red line) on the monophenolase activity of mushroom tyrosinase.

The samples that inhibited the monophenolase activity of tyrosinase were measured using L-tyrosine as the substrate. In the reaction system, OR was included for comparison. Fig. 7 shows the effects of different concentrations of MA on the inhibition of the monophenolase activity of mushroom tyrosinase. As shown in Fig. 6, in a certain concentration range (0∼3.5 µmol/L), the inhibition rate increased with increased concentrations of the sample. However, there were differences in the inhibitory activity between the two substances. MA and OR resulted in similar inhibition. The IC_50_ values of MA and OR were 1.29 µmol/L and 0.12 µmol/L, respectively. These results indicated that MA and OR strongly inhibited the monophenolase activity of the mushroom tyrosinase. The inhibitory activity of OR was approximately 10-fold stronger than that of MA, which indicated that the two glucose molecules added to OR had some effect on the inhibition of the monophenolase activity of the mushroom tyrosinase.

### 3.9 The inhibition mechanism of MA on the monophenolase activity of tyrosinase

**Figure 8:**
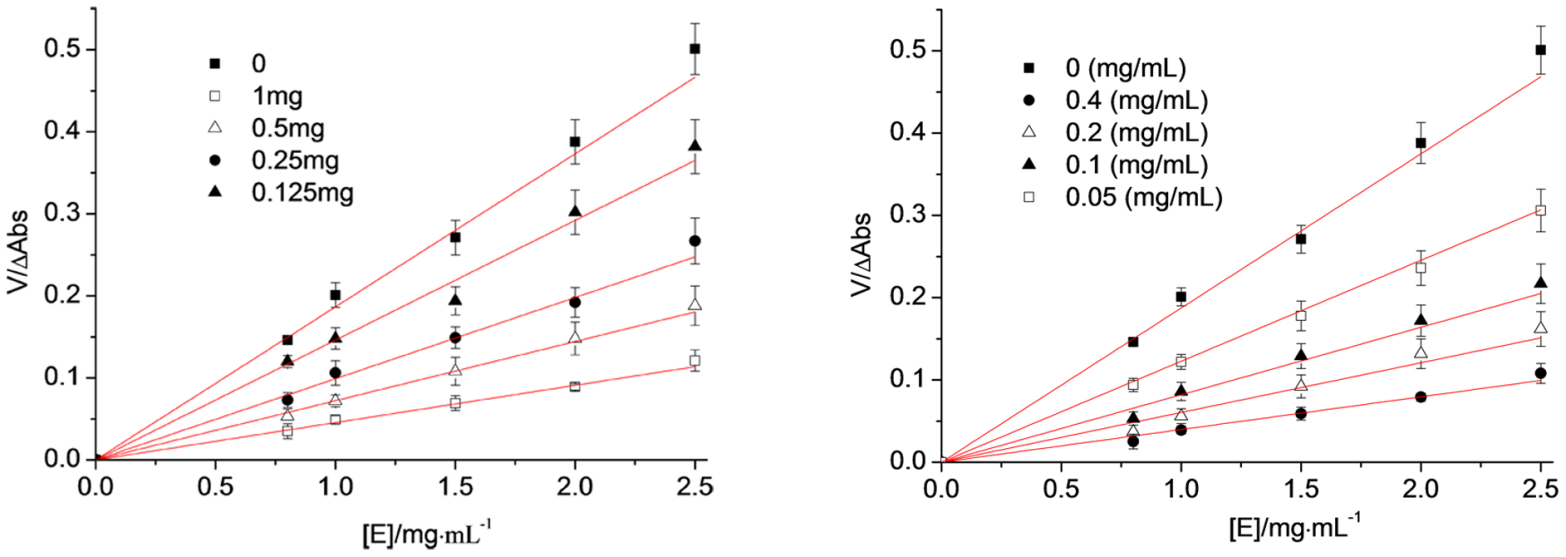
The reversible inhibitory reaction of Mulberroside A (Left) and oxyresveratrol (Right) on monophenolase of mushroom tyrosinase.

By adding different concentrations of samples to the reaction system, when the concentration of L-tyrosine was unchanged, but the amount of enzyme added was changed, the reaction velocity (V) of the enzyme-catalysed oxidation of the substrate was measured by the change in the amount of enzyme ([E]). OR was the control in this test, which measured the mechanism by which MA inhibits the monophenolase activity of tyrosinase. The selected sample concentrations of MA were 0, 0.22 mmol/L, 0.44 mmol/L, 0.88 mmol/L and 1.76 mmol/L; the sample concentrations of OR were 0, 0.21 mmol/L, 0.42 mmol/L, 0.84 mmol/L and 1.68 mmol/L; and the enzyme concentrations were 0.8 mg/mL, 1.0 mg/mL, 1.5 mg/mL, 2.0 mg/mL and 2.5 mg/mL. The results are shown in Fig. 8. A group of straight lines through the origin was obtained for both MA and OR in the system, and the slope of the line decreased with increased concentrations of the samples, which indicated that the inhibitory effect of MA and OR on the monophenolase of the mushroom tyrosinase was a reversible process. In addition, the effects of MA and OR on the activity of the enzyme were caused by the inhibition of enzyme activity rather than irreversible inactivation. Therefore, both MA and OR were reversible inhibitors of the mushroom tyrosinase.

### 3.10 The inhibition constant of MA on the monophenolase activity of tyrosinase

**Figure 9:**
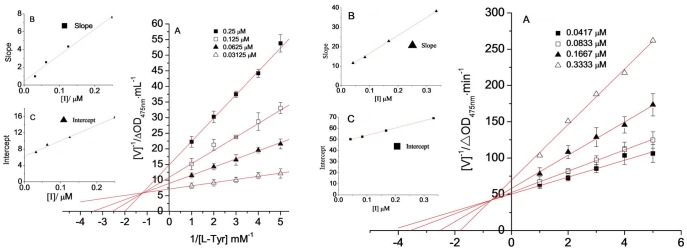
Determination of the inhibitory type and inhibition constant of MA (Left) and oxyresveratrol (Right) on monophenolase of mushroom tyrosinase. (A) Lineweaver-Burk plots for inhibition of MA or oxyresveratrol on monophenolase. (B) and (C)represent the plots of slope and intercept versus the concentration of MA for determining the inhibition constant KI and KIS.

The concentration of enzyme in the reaction system was constant. The impact of different concentrations of samples on enzyme activity was measured by changing the concentration of L-tyrosine and using Lineweaver-Burk double reciprocal plots. The reciprocal of the substrate concentration 1/[S] was the *x*-axis, and the reciprocal of the reaction rate 1/V was the *y*-axis. The results are shown in Fig. 9. A group of straight lines intersected in the second quadrant. This result illustrated that increased sample concentrations not only led to a decrease in the maximum reaction velocity (*V_max_*) of the enzymatic reaction but also to an increase in the Michaelis-Menten constant (*K_m_*). These results showed that MA inhibited the monophenolase of the mushroom tyrosinase by a mixed-type inhibitory pattern, which was identical to OR. The inhibition constants of MA and OR on free enzyme (*K_I_*) and on the enzyme-substrate complex (*K_IS_*) were obtained by plotting the slope and the *y*-axis intercept against the sample concentration (Table 1), respectively.

**Table 1:** Inhibition effect of mulberroside A and of oxyresveratrol on the monophenolase activity of the mushroom tyrosinase.

Sample	IC_50_ (µmol/L)	Inhibition mechanism	Inhibition type	*K_I_* (µmol/L)	*K_IS_* (µmol/L)
MA	1.29	reversible	mix-type I	0.385	0.177
OR	0.12	reversible	mix-type I	0.926	0.662

### 3.11 The inhibition of MA on the diphenolase activity of tyrosinase

**Figure 10:**
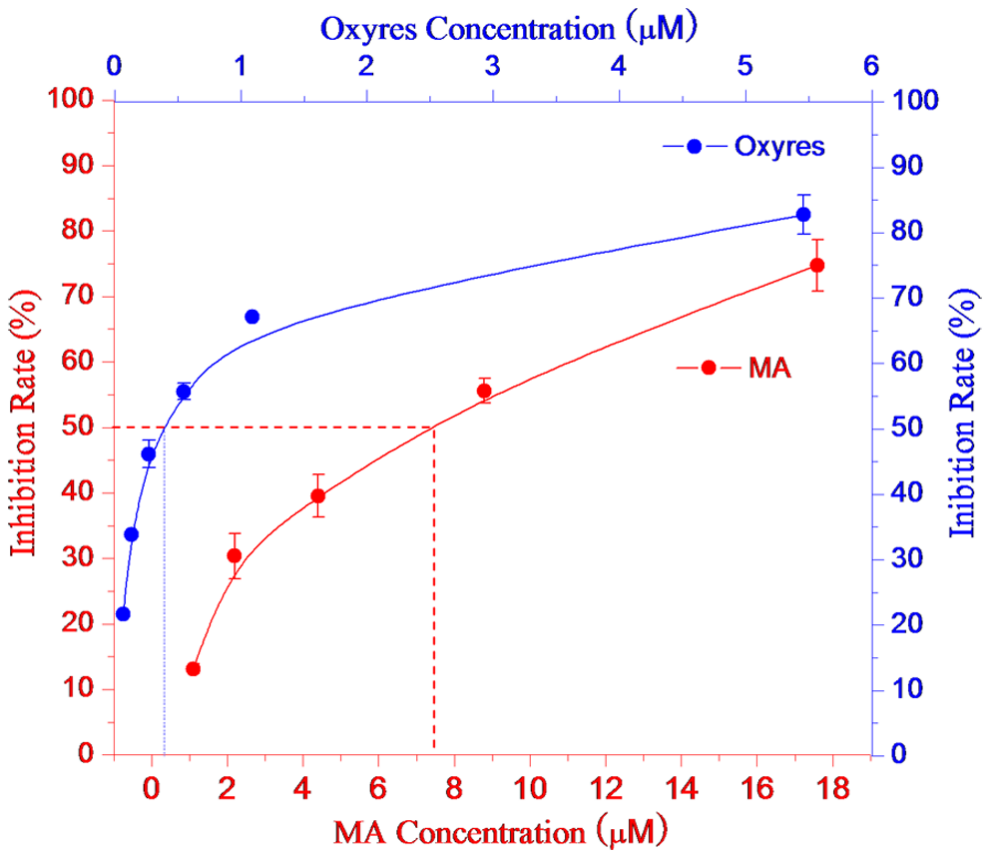
Inhibition effects of mulberroside A (blue) and oxyresveratrol (red) on the diphenolase activity.

The samples that inhibited the diphenolase activity of tyrosinase were measured with L-DOPA as the substrate. Different concentrations of MA or OR were added to the reaction system to monitor their effects on the diphenolase activity of the mushroom tyrosinase. The results are shown in Fig. 10. In a certain concentration range, in which the inhibition rate increased with increased sample concentrations. However, there were differences between MA and OR on the inhibition of diphenolase activity. The IC_50_ values of MA and OR were 7.45 µmol/L and 0.39 µmol/L, respectively. These results showed that OR had the strongest activity on diphenolase, whereas MA was weaker. Compared with MA, the IC_50_ value of OR was 19-fold lower. This result indicated that the inhibition of diphenolase was weakened after the two glucose molecules were added to OR.

### 3.12 The inhibition mechanism of MA on the tyrosinase diphenolase

**Figure 11:**
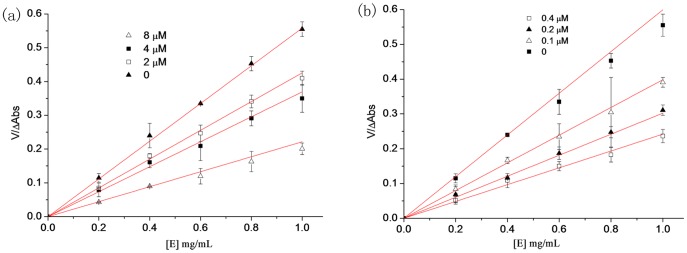
The inhibitory mechanism of Mulberroside A (a) and oxyresveratrol (b) on diphenolase of mushroom tyrosinase was reversible.

The concentration of the substrate, L-DOPA, was unchanged in the reaction system; however, the amount of enzyme added was changed. The relation between the velocity of the enzyme-catalysed oxidation of the substrate (V) and the amount of enzyme ([E]) was measured after adding different sample concentrations. The concentrations of MA were 0, 2.0 µmol/L, 4.0 µmol/L and 8.0 µmol/L; the concentrations of OR were 0, 0.1 µmol/L, 0.2 µmol/L and 0.4 µmol/L; and the enzyme concentrations were 0.2 mg/mL, 0.4 mg/mL, 0.8 mg/mL and 1.0 mg/mL. A plot of absorbance change against the amount of enzyme (Fig. 11) showed that both MA and OR resulted in a group of straight lines through the origin, and the slope of the lines decreased with increased sample concentrations. These results illustrated that the inhibition mechanisms of MA and of OR on the diphenolase activity was a reversible process and that both MA and OR inhibited the diphenolase activity but did not lead to irreversible enzyme inactivation. Therefore, these experimental results further showed that both MA and OR were reversible inhibitors of the mushroom tyrosinase. However, the differences in IC_50_ values between MA and OR showed that OR was the stronger reversible inhibitor, whereas MA was the weaker inhibitor.

### 3.13 The inhibition constant of MA on the diphenolase activity of tyrosinase

**Figure 12:**
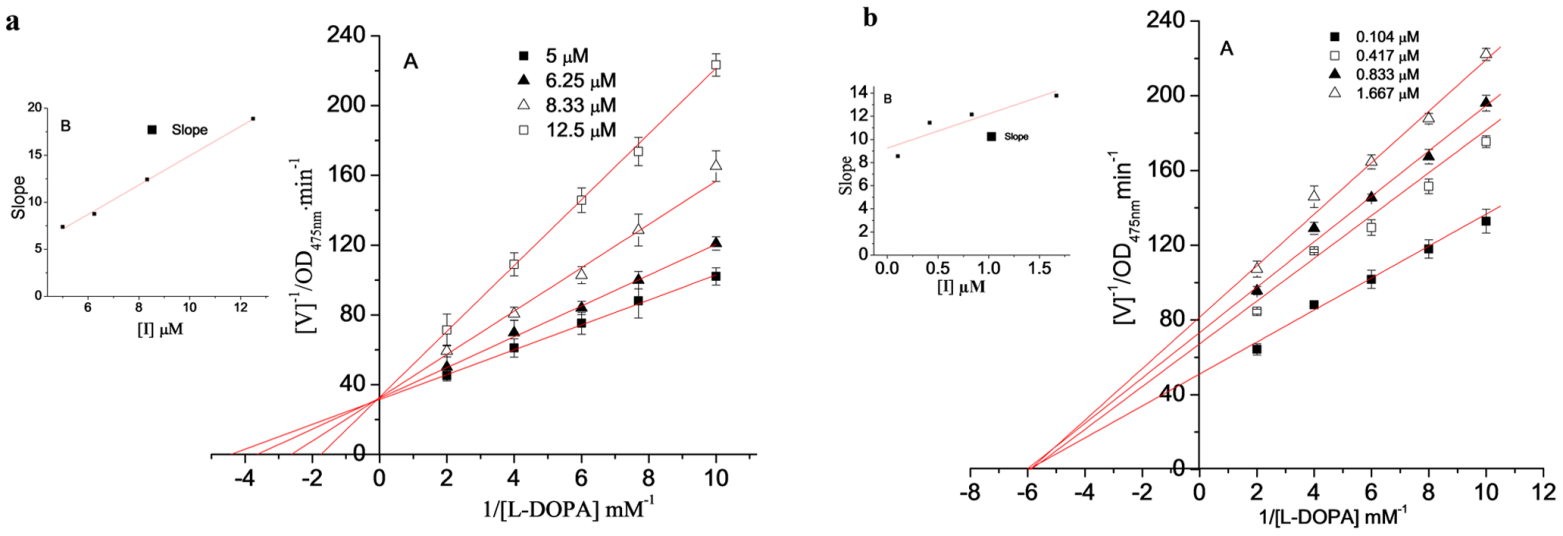
Inhibition constant of MA (a) and oxyresveratrol (b) on diphenolase of mushroom tyrosinase. (A) Lineweaver-Burk plots for inhibition of MA on diphenolase. (B) The plots of slope versus the concentration of mulberroside A for determining the inhibition constants KI, respectively.

The concentration of the enzyme in the reaction system was constant. The impact of different concentrations of samples on the enzyme activity was measured by changing the concentration of L-DOPA and using Lineweaver-Burk double reciprocal plots ^6^. The reciprocal of the substrate concentration 1/[S] was the *x*-axis, and the reciprocal of the reaction rate 1/V was the *y*-axis. The results are shown in Fig. 12. The double reciprocal plots of MA and of OR showed significantly different results. A group of lines that intersected on the *y*-axis was obtained in the plot of MA, which indicated that the maximum reaction velocity (*V_max_*) did not change when the sample concentration increased; however, the Michaelis-Menten constant (*K_m_*) increased. This result indicated typical competitive inhibition. The lines obtained by the double reciprocal plot of OR intersected on the *x*-axis, which indicated that the Michaelis-Menten constant (*K_m_*) was not affected by increased sample concentrations but led to decreased maximum reaction velocity (*V_max_*). This result indicated non-competitive inhibition. Secondary plots of the slope and *y*-axis intercept of each line against sample concentration were obtained to determine the inhibition constants of MA on the free enzyme (*K_I_* = 4.36 µmol/L) and OR on the enzyme-substrate complex (*K_IS_* = *K_I_* = 2.95 µmol/L) (Table 2).

**Table 2:** Inhibition effects of MA and of OR on the diphenolase activity of the mushroom tyrosinase.

Sample	IC_50_ (µmol/L)	Inhibition mechanism	Inhibition type	*K_I_* (µmol/L)	*K_IS_* (µmol/L)
MA	7.45	reversible	competitive	4.36	—
OR	0.39	reversible	Non-competitive	2.95	2.95

—: none.

## Discussion

Tyrosinase is a polyphenol oxidase and is not only the key enzyme in the food browning reaction [21] but is also involved in mammalian melanin formation [22]. Enzymatic browning in fresh fruits and vegetables usually leads to the loss of nutrition [23]. Thus, the use of tyrosinase inhibitors appears to be a promising approach to suppress undesirable browning reactions to help maintain the quality of food products and can be used as an important additive in whitening products in the cosmetics industry. Well-known examples include arbutin, kojic acid and glabridin. These compounds have good inhibitory effects; however, their uses are limited and, therefore, remain not widely used. Therefore, the identification of a tyrosinase inhibitor that has a wide range of sources, a good inhibitory effect and no adverse effects has become a focus of research in recent years.

Concerning the molecular structure analysis, the hydrogen of the two phenolic hydroxyls at the 4′ and 3′ position in OR were substituted by two glucose molecules, which slightly affected the activity of MA in inhibiting the monophenolase activity of tyrosinase, and the IC_50_ values only had 10-fold differences. However, both of these hydroxyls had strong inhibition that is a mixed inhibition type. However, the presence of the two glucose molecules had a significant effect on the inhibition of the diphenolase activity of the tyrosinase. The IC_50_ value of MA was only 7.45 µmol/L and was almost 20 times that of OR. The inhibition types of MA and OR on the diphenolase activity were different. These findings illustrated that the presence of the two glucose molecules seriously affected the diphenolase inhibitory activity.

Our results were both similar and different when compared with the previous findings reported by others. The results concerning the effect of OR on the inhibition of the mushroom tyrosinase were similar to our results. The strong tyrosinase inhibitory activity of OR was previously confirmed in other studies, which found that the IC_50_ value of OR was 0.46∼1.10 µmol/L, depending on the assay conditions [8], [24] Concerning the OR-inhibited monophenolase activity, the IC_50_ value of OR extracted from the mulberry twigs was 0.943 µmol/L (0.23 µg/L), which was reported by Lee et al. [8]. Compared with our results, there was an 8-fold difference in the IC_50_ value. The IC_50_ values of OR, which inhibited the monophenolase and diphenolase activities, were approximately 4-fold and 30-fold lower than that of our experiment, respectively. Their inhibition mechanism and type were also identical [6]. However, the IC_50_ value of MA for the inhibition of the monophenolase activity was almost 40-fold lower than our results, and the inhibition type was different. Kim's results showed competitive inhibition, whereas our results showed mixed-type inhibition. In addition, our results showed that MA had apparent inhibition of the diphenolase, which differed from their reports [24]. These inconsistent results were most likely caused by differences in the purity of the sample, the source of the mushroom tyrosinase, the reaction systems and operator errors. We continue to thoroughly examine these issues.

MA has a strong inhibitory activity on tyrosinase. However, compared with OR, which has a similar structure, there are few reports concerning the biological activity and function of MA. MA also has poor permeability [24]. Nevertheless, a pharmacokinetic analysis in 1996 showed that a small number of MA derivatives were found in the blood of rats after oral administration of the mulberry root acetone extract, and the bioavailability was only approximately 1%. This result was because most of the MA was transformed to OR and transferred into the circulating blood, and the absorption rate was estimated to be 50%. OR was also rapidly transported into the tissues [3]. Three major metabolites were isolated from rat faeces, including oxyresveratrol-2-*O-β*-D-glucoside, oxyresveratrol-3′-*O-β*-D-glucoside and OR. This result indicated that MA was metabolised before being absorbed [25]. Following incubation with intestinal bacteria under anaerobic conditions, MA was rapidly deglucosylated and transformed into OR or generated aglycon-oxyresveratrol [26]. OR has anti-bacterial, anti-inflammatory [26], and neuroprotective [27] activities and can repair liver injury induced by alcohol [28]. Therefore, MA used as an oral preparation can be converting into OR *in vivo*. This conversion into OR can increase its permeability and then can easily be absorbed by cells, which can use a variety of physiological activities of OR. Thus, the pharmacological effects of MA should be studied together with OR to obtain in-depth knowledge concerning these compounds.

Although Wu [5] et al. obtained 1.346% MA using ultrasonic extraction, the MA was extracted from the root, and the method was extremely complicated. From the analysis of our experiments, we can efficiently extract 0.412% of oxyresveratrol diglycopyranoside from waste mulberry branches in the sericulture industry using the method reported in this article. The extract also showed strong tyrosinase inhibitory activity. Therefore, this method can be used in the reclamation of MA from large mulberry biological resources, which can not only be widely used in whitening cosmetics but also have important development potential in anti-oxidative and anti-aging biological drugs, as well as in health foods.

## Conclusions

In this study, only D101 macroporous resin and a semi-preparative HPLC system were used to efficiently extract MA from mulberry twigs. The purity of MA was as high as 98.3%. The enzymatic analysis showed that MA and OR had strong inhibitory activities on the mushroom tyrosinase. In vitro, MA inhibited the synthesis of dopachrome primarily by inhibiting the two steps of the first process, which inhibited tyrosinase activity, and had weaker inhibitory activity on diphenolase. However, MA had no effect on the non-enzymatic process.

Tyrosinase inhibitory activity analyses showed that MA resulted in strong inhibition of the monophenolase activity of the mushroom tyrosinase. Additionally, the IC_50_ value was 1.29 µmol/L, which had a significant difference compared with the inhibitory activity of OR (IC_50_: 0.12 µmol/L). Compared with OR (IC_50_: 0.39 µmol/L), MA showed weaker inhibition of the diphenolase of the mushroom tyrosinase, and the IC_50_ value was 7.45 µmol/L. The analyses of the inhibition mechanism of the monophenolase and diphenolase of the tyrosinase indicated that MA showed reversible inhibition, which was similar to OR. The inhibition activities of MA and of OR on the monophenolase of tyrosine was mixed-type 1. Additionally, the inhibition constants *K_I_* were 0.385 µmol/L and 0.926 µmol/L, respectively, and *K_IS_* values were 0.177 µmol/L and 0.662 µmol/L, respectively. For diphenolase activity, there were some differences between MA and OR. MA showed competitive inhibition, and K_I_ was 4.36 µmol/L; however, OR showed noncompetitive inhibition, and *K_I_ = K_IS_ = *2.95 µmol/L.

## Supporting Information

Figure S1 **1H-NMR (CD3OD, 300 MHz) spectrum of the MA.**
(PDF)

Figure S2 **13C-NMR (CD3OD, 75 MHz) spectrum of the MA.**
(PDF)

Figure S3 **1H-NMR (CD3SOCD3, 400 MHz) spectrum of the MA.**
(PDF)
