# Supplementary figures and images for: An Efficient Preparation of Mulberroside A from the Branch Bark of Mulberry and Its Effect on the Inhibition of Tyrosinase Activity
Source: PLoS One. 2014 Oct 9;9(10):e109396. doi: 10.1371/journal.pone.0109396 (PMC4192315; doi:10.1371/journal.pone.0109396)

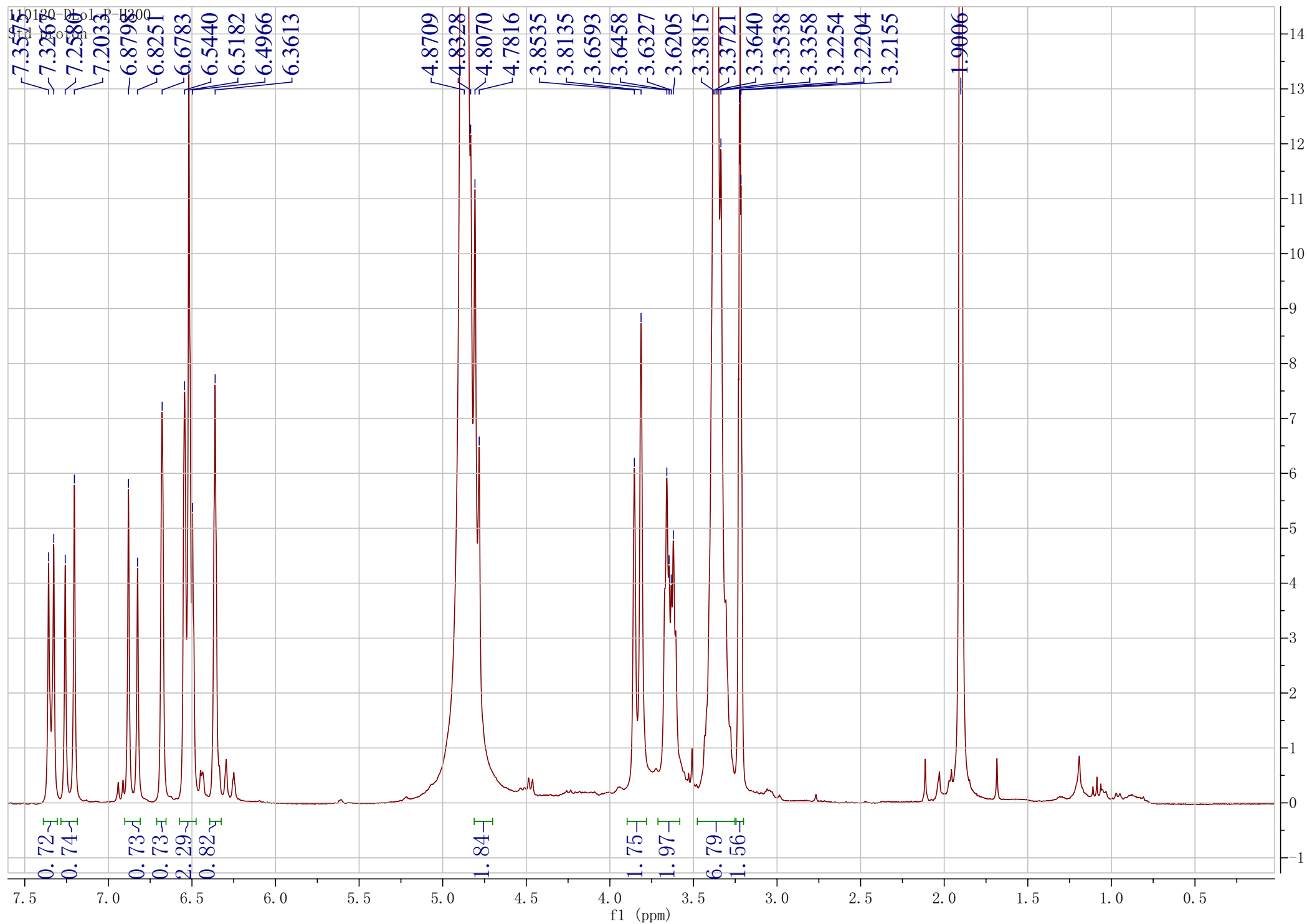

Supplement: Figure S1 — 1H-NMR (CD3OD, 300 MHz) spectrum of the MA. (PDF) [file pone.0109396.s001.pdf]

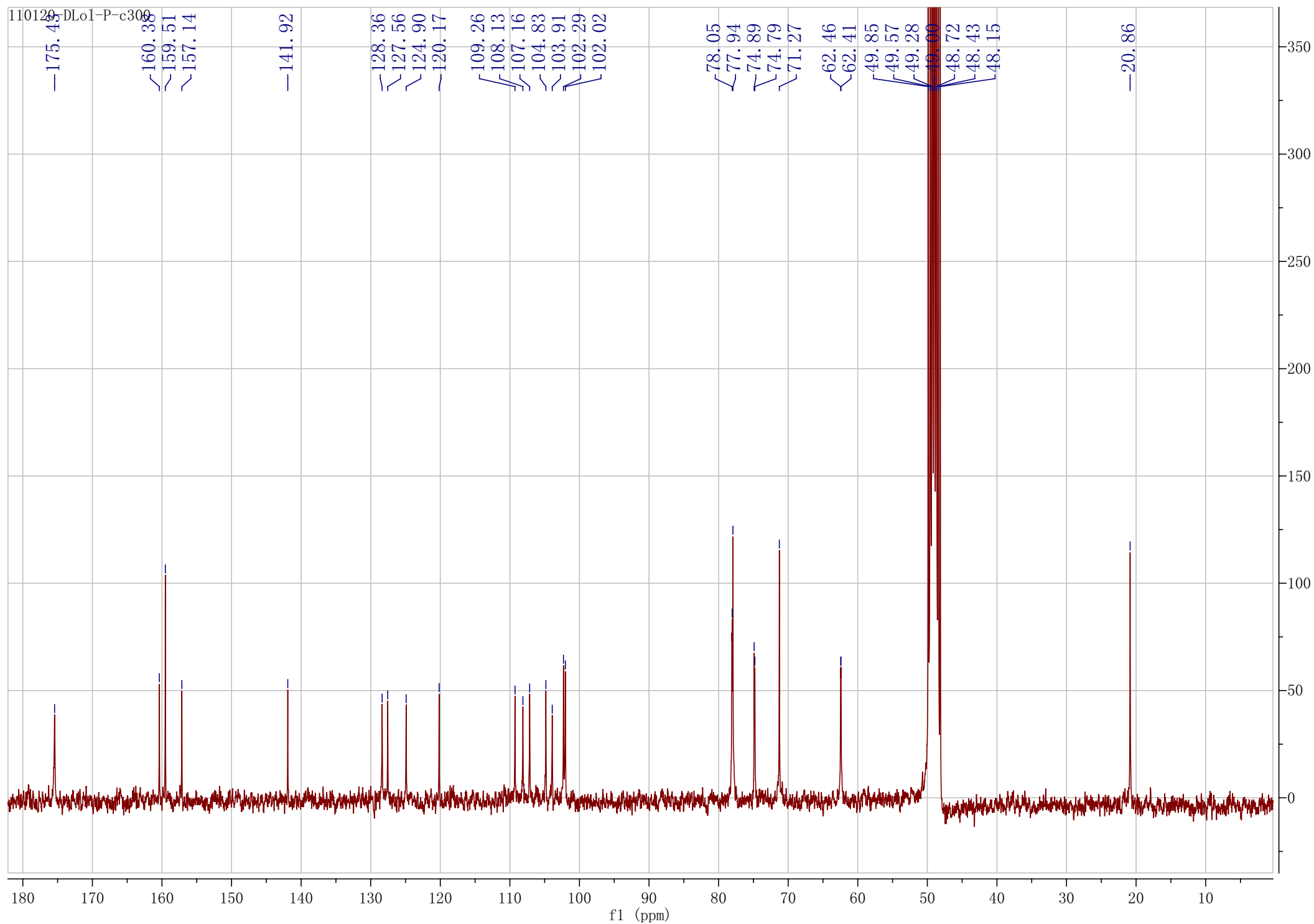

Supplement: Figure S2 — 13C-NMR (CD3OD, 75 MHz) spectrum of the MA. (PDF) [file pone.0109396.s002.pdf]

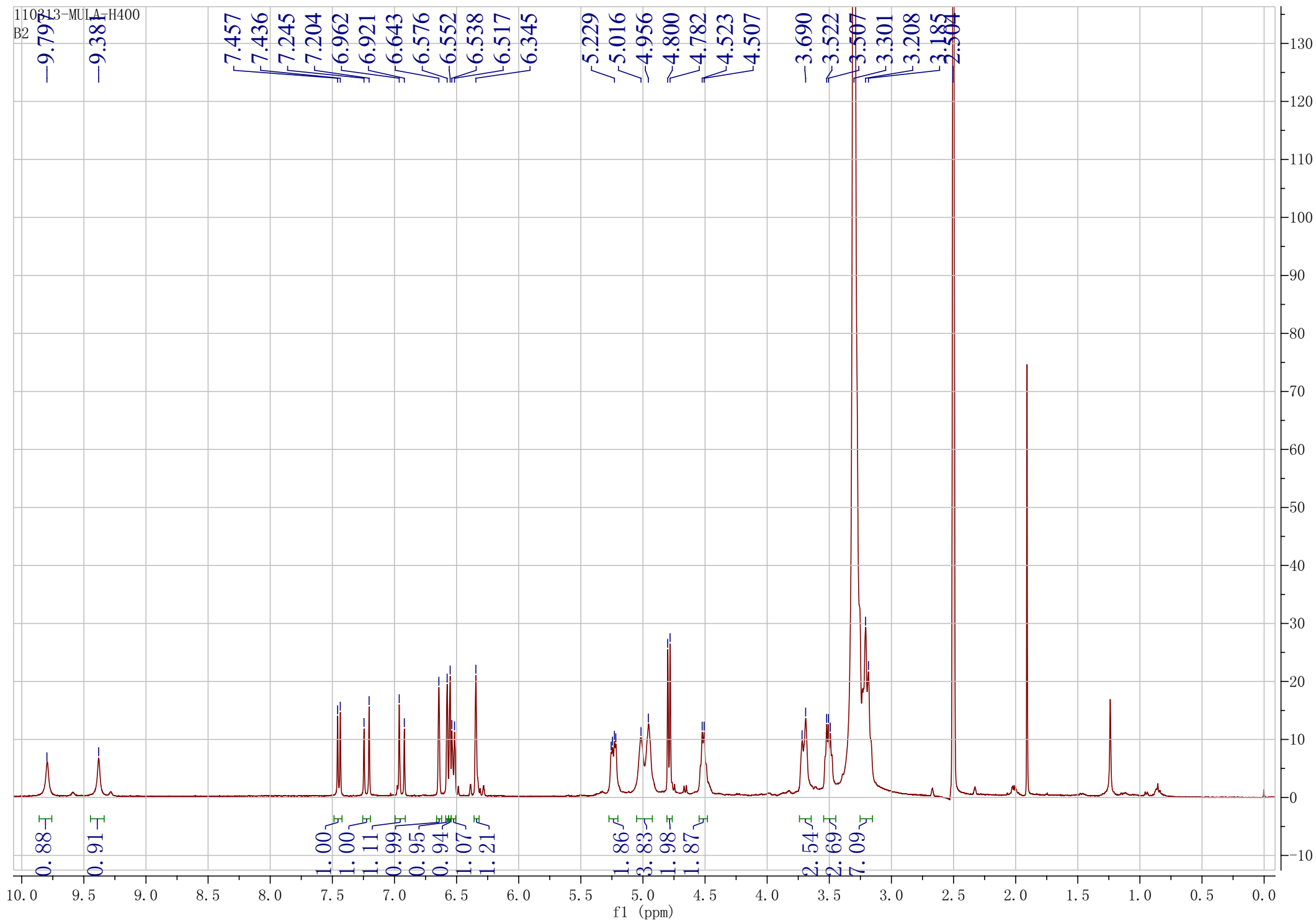

Supplement: Figure S3 — 1H-NMR (CD3SOCD3, 400 MHz) spectrum of the MA. (PDF) [file pone.0109396.s003.pdf]
